# Adolescents’ Tsunami Exposure and Mental Health Consequences: Protective Role of Cultural Coping Strategies

**DOI:** 10.3390/ijerph21060756

**Published:** 2024-06-09

**Authors:** Thulitha Wickrama, Michael J. Merten, K. A. S. Wickrama, Amanda Terrell

**Affiliations:** 1 Department of Child, Youth and Family Studies, University of Nebraska-Lincoln, Lincoln, NE 68588, USA; michael.merten@unl.edu; 2 Department of Human Development and Family Science, University of Georgia, Athens, GA 30602, USA; wickrama@uga.edu; 3 School of Human Environmental Sciences, University of Arkansas-Fayetteville, Fayetteville, AR 72701, USA; amandat@uark.edu

**Keywords:** disasters, adolescence, mental health, cultural coping, developing countries, tsunami, trauma

## Abstract

There is a knowledge gap regarding the link between disaster exposure and adolescent mental health problems in developing countries. This study examines the case of Sri Lanka to investigate (a) the immediate and long-term mental health impact of the 2004 tsunami disaster on adolescents and (b) the potential moderating effects of unique cultural and family practices that prevail in Sri Lanka. This study used a random sample of 160 adolescents (ages 12–19) and their mothers who were exposed to the tsunami disaster while living in a southern Sri Lankan village and provided prospective data immediately after the disaster (2005) and three years later (2008). A cross-culturally validated instrument assessed adolescent–mother dyads’ tsunami exposure, stressful loss, family cultural rituals and familism, and adolescent mental health. Structural equation modeling analysis showed that exposure and perceptions of tsunami-induced stressful experiences were associated with early and later mental health problems in adolescents. In addition, this study found that unique cultural practices and familism moderated the link between adolescent tsunami exposure, stressful experiences, and levels of PTSD and depressive symptoms. The findings of this study could be utilized to develop prevention and intervention programs that are contextually and culturally valid and empirically supported, which would be more effective for trauma-exposed adolescents in developing countries.

## 1. Introduction

Developing countries are more exposed and more vulnerable to natural disasters compared with developed countries [1]. Previous studies have highlighted the crucial point that the mental health impact of natural disasters is intensified in developing countries due to adverse contexts, such as poor-quality housing, weakened infrastructure, existing war zones, absence of warning systems, and insufficient resources needed for recovery [2,3,4]. Thus, a natural disaster such as a tsunami more severely effects the health, resources, and livelihoods of those in developing countries compared to those in a more developed country. The impacts of disasters have shown to be ten times worse on the economies of developing countries compared to more developed countries [5]. In addition to direct exposure to natural disasters, disaster-related stressful life experiences also influence mental health [6]. Previous research indicates that the severity of traumatic disaster exposure (e.g., property destruction, social/family losses, and witnessing deaths and injuries) and related stressful experiences (family, social, and instrumental support losses) can lead to both post-traumatic stress disorder (PTSD) and depression [3,7]. Exposure to multiple traumatic and stressful experiences may also intensify the mental health consequences of exposure to any single traumatic or stressful experience [7,8,9].

Most previous disaster studies have overwhelmingly focused on adults’ mental health consequences related to disaster experiences [3,7,9,10,11], and the research conducted on children and adolescents in developing countries to investigate their mental health consequences is insufficient [12,13]. In adolescence, disaster experiences may not only lead to early mental health problems but also long-term health consequences [14,15,16]. Most previous studies have not examined such long-term mental health consequences in adolescents. This lack of knowledge regarding the association between disaster exposure and adolescent mental health consequences in developing countries serves as the motivation for this current study, which aims to provide a deeper longitudinal investigation into the mental health consequences for disaster-exposed adolescents. The first objective of the present study was to investigate early and long-term mental health consequences of natural disasters for adolescents in developing countries.

Studying adolescent mental health in developing countries is crucial due to the significant proportion of young people in these regions who face extreme challenges related to poverty and limited healthcare access that deteriorates mental health [17]. While these challenges also face vulnerable youth in more developed countries, they are experienced more severely, in more difficult environments lacking infrastructure, and with fewer accessible and quality supportive resources. In fact, UNICEF [18] describes youth who face both chronic and acute conflict and crisis as the most at risk (e.g., experiencing a natural disaster in the context of a war zone). There is a scarcity of research on these youth, particularly on understanding how cultural contexts can lead to more effective, localized mental health strategies and early interventions to prevent long-term problems, benefiting overall societal and economic health [17].

Previous disaster research suggests that the majority of people exposed to natural disasters are resilient and recover from early psychological symptoms [3,19,20]. Importantly, several studies have identified various psychosocial resources, particularly unique family, cultural, and religious practices in developing countries, that may protect disaster-exposed victims from short-term and long-term mental health consequences. However, these studies have overwhelmingly focused on the disaster-exposed adult population. While parents and families are clearly central to children’s resiliency following disasters [8], less is known about the role of family and cultural practices in developing countries that could protect disaster-exposed adolescents from mental health consequences [21,22]. Thus, the second objective of this study is to investigate the potential moderating role of unique family and cultural practices that may protect natural disaster-exposed adolescents in developing countries from mental health consequences.

### 1.1. The 2004 Tsunami

The study objectives were investigated in the context of the 2004 tsunami disaster in Sri Lanka. Most people in Sri Lanka are engaged in traditional agricultural and fishery livelihoods within their communities [23]. Families living in these communities are closely linked through traditional collective, sharing, and supporting social networks at the grassroots level [9]. Parents and children in these families maintain high levels of support among themselves [24]. Sri Lanka has higher human development indicators compared to other developing countries, including literacy rate (more than 90%), life expectancy (76.4 years), infant mortality (5.8 death per 1000 live births), and maternal mortality (30.2 per 100,000 live births). These indicators reflect the political, social, and educational contexts in which the adolescents and families of this study are embedded [25,26].

**Figure 1:**
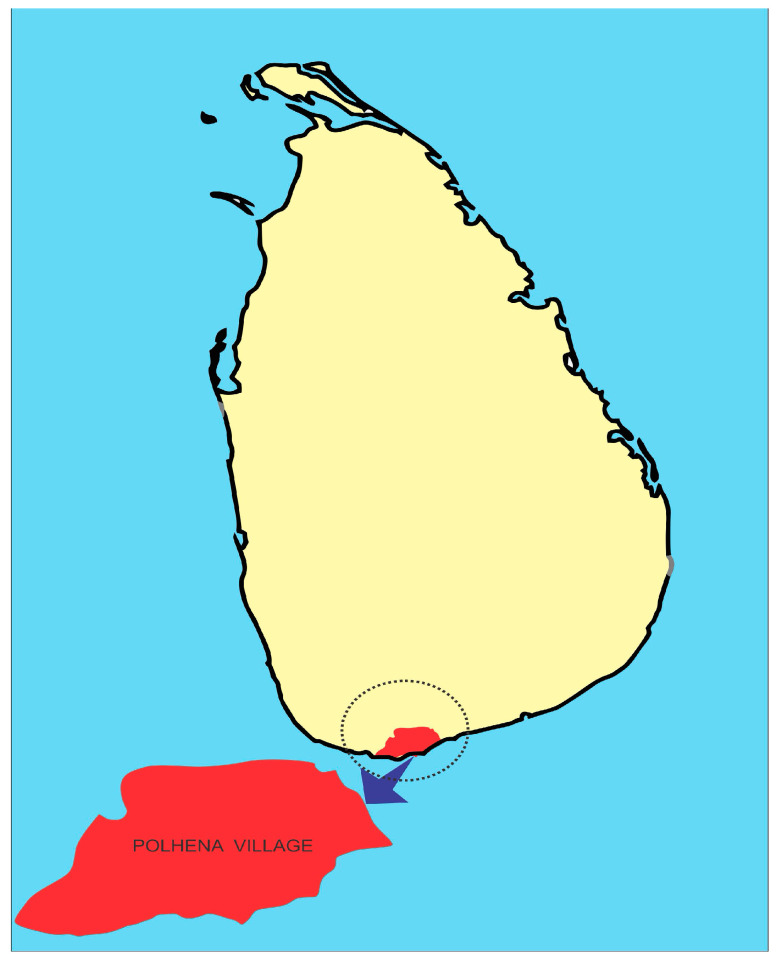
Author-drawn map of the southern Sri Lankan village of Polhena, the data collection site for the current study.

On 26 December 2004, a tsunami claimed 31,000 lives in Sri Lanka and left more than 100,000 people homeless [27]. The tsunami had a severe impact on millions of families in the area, resulting in the destruction of their homes, communities, and damaging social and physical resources. Additionally, millions of children and adults experienced traumatic events and stressful experiences as a result of the tsunami. This study utilizes longitudinal data from families residing in a southern Sri Lankan village (Polhena) that were collected in early 2005 (less than three months after the tsunami) and again in 2008 (Figure 1). This study explored relations between the level of tsunami exposure/experiences, levels of PTSD and depressive symptoms in adolescents, and familial and cultural practices.

### 1.2. Theoretical Background

We draw our theoretical framework from the stress process perspective [28] and stress appraisal theory [29], which posit that stressful life experiences have mental health consequences for individuals and that this association may be moderated by available psychosocial and cultural resources that support resiliency. We contend that for adolescents, these resources may include participation in family cultural practices because both children and parents participate in these cultural practices. We will discuss these hypothesized associations in the following subsections.

## 2. Literature Review

### 2.1. Adolescents’ Disaster-Related Stressful Experiences and Depressive Symptoms

Stress appraisal theory [29] illustrates that when an individual is exposed to a stressor, he/she subjectively appraises the seriousness and threat of the stressor and assesses the resources available to manage the stressor. Accordingly, disaster-exposed individuals may experience feelings in their post-disaster environment such as hopelessness, disbelief, helplessness, loneliness, uncertainty, sadness, and insecurity. This is because such an environment is often characterized by a loss of social support, facilities/opportunities, benefits, and other negative factors [30]. This intra-individual process often leads to an escalation of negative feelings. This perceived stress can lead to above-average levels of depressive symptoms. Accordingly, tsunami-related stressful experiences, such as social and family losses, are expected to elevate depressive symptoms in adolescents.

### 2.2. Adolescents’ Exposure to Traumatic Events and PTSD Symptoms

Previous research has indicated that traumatic events, such as serious injuries, property destruction, and witnessing deaths, predict PTSD symptoms through cognitive re-experiences, avoidance, and hyperarousal processes [3,31,32]. Cognitive re-experiencing includes things such as “continued thinking about the event, nightmares, vivid flashbacks, trouble sleeping/concentrating, and intrusive thoughts or memories. Avoidance/numbing includes experiences such as attempting to avoid thinking or talking about the event and being unable to feel happy, sad, or excited about things. Conditions of hyperarousal include feeling jumpy or being startled by a sudden noise or movement” [33]. We expect to capture the overall level of adolescents’ PTSD symptoms using 17 diagnostic interview items from the DSM-IV [33].

### 2.3. Protective Role of Cultural Practices

It is imperative that disaster research also focus on factors and processes that promote proactive healing and resiliency. Previous disaster studies [28,34,35] have suggested that adaptive systems [7] like social and cultural resources and practices can protect trauma/stress-exposed individuals from adverse mental health consequences. Although most families in tsunami-affected communities were exposed to the disaster, they may have possessed unique social and cultural resources that helped them better recover/cope from serious trauma [36,37]. However, only a handful of studies have been directed toward culturally specific healing practices and rituals that individuals and families may use to cope with traumatic events and stress following disasters [38]. Despite the centrality of families, even fewer studies have focused on family coping strategies that may be effective for disaster-exposed adolescents. Our previous research provided empirical evidence of Sri Lanka’s cultural coping methods, moderating the association between tsunami disaster exposure and mental illness among mothers over time [39]. Integrating indigenous belief systems including family coping strategies can improve the effectiveness of standard Western disaster recovery interventions [40,41,42].

In Sri Lanka, popular cultural rituals that are used to cope with mental health problems mainly include the Bali, Thovil, and Atanatiya rituals. These rituals are proactive coping strategies that involve resource investments to buffer the effects of resource loss that may lead to distress [43]. The objective of these rituals is to eliminate “beings” with superhuman powers that cause mental illness in spiritual form. Cultural ritual practices involve astrology, spirits, the deities associated with them, and their ethics, morality, and beliefs.

*Bali Rituals* are conducted to help mentally ill individuals recover by driving away evil spirits, or Buthayo, inhabiting the mind and body of the patient. This elaborate ritual is conducted by a Bali performing artist. He will dance, drum, and recite spiritual stanza integrated with Buddhist philosophy. It is expected that the belief of Buddha as a superior spiritual power will act to heal the mentally ill patient. Devil dancing, or the *Thovil Ritual*, is a demonic exorcism ritual commonly practiced and gaining in popularity in Sri Lanka. In this practice, there is a belief that mental illness in a person is evoked by the devils or Yakkas who have entered the mind and body of the mentally ill individual [44,45]. The Thovil ritual ceremony is conducted to rid the individual of these Yakkas and heal the patient. Yakkas, accepted to be part of the natural universe, are supposed to follow their leader Vesamuni, a fervent follower of Buddhism. The evidence of the Yakkas leaving the ill person is found when there is a breaking of a branch or a loud sound at the demand of the ritual conductor or Kattadiya. This sound is claimed to be audible to participants, many of whom strongly believe in culturally traditional explanations and cures for mental illness. While Bali and Thovil are not part of Buddhist doctrine, they refer to Buddhist teachings.

The *Atanatiya Ritual* is considered a purely Buddhist form of an exorcist ceremony that Sri Lankans have been practicing for millennia to treat mental illness [44]. According to Kariyawasam [44], it is perhaps the oldest used exorcism-based ritual, but it is less common. The ritual heavily incorporates structural elements of Buddhism, with a Buddhist monk reciting Buddhist scripture in certain instances, often within the temple premises, to drive away the evil spiritual influence of the Yakkas from a mentally ill or possessed person. Spiritual influence is exorcised through the power of good karma.

Dissociative coping may reduce mental illness symptoms while engaging in cultural coping [46,47,48]. Cultural coping strategies also provide social support from friends, family, and community for reaffirming the cultural beliefs, that aids in reducing mental illness [49]. For example, in southern Sri Lanka, where people were affected by the tsunami, family and community members often organize rituals for the benefit of distressed individuals and the community as a whole. The organization of cultural beliefs and rituals to address symptoms engages not only distressed individuals but also their family and community members in this process [7,38]. This is achieved by involving the family and community members in the ritual activities as audience members, dancing, singing, and providing support to the patient during the ritual. Their participation, a devotional activity and caregiving activity, exerts direct and indirect social support to the patient.

### 2.4. Protective Role of Family Practices

Familism is the seeking, receiving, and providing of support to family members, and it has been shown to reduce mental illness [23,50]. In traditional societies like Sri Lanka, where families typically exhibit high levels of familism, adolescents may be protected from the negative effects of tsunami-related losses through direct compensatory mechanisms involving familism. We expect that high levels of familism should be related to decreased levels of PTSD and depressive symptoms among mothers who had experienced the tsunami.

It is important to understand culturally specific beliefs, behaviors, values, and philosophies, as well as their dynamics, within families and society through a social, psychological, and anthropological lens. This understanding can inform post-disaster interventions, leading to improved effectiveness and sustainability [51]. Such an approach can help bridge the gap between the seemingly opposite traditional beliefs centered on karma and samsara in Hindu and Buddhist cultures in the East and the Western psychological emphasis on introspection, behavior, choice, causality, and targeted interventions [52].

### 2.5. Present Study and Specific Hypotheses

**Figure 2:**
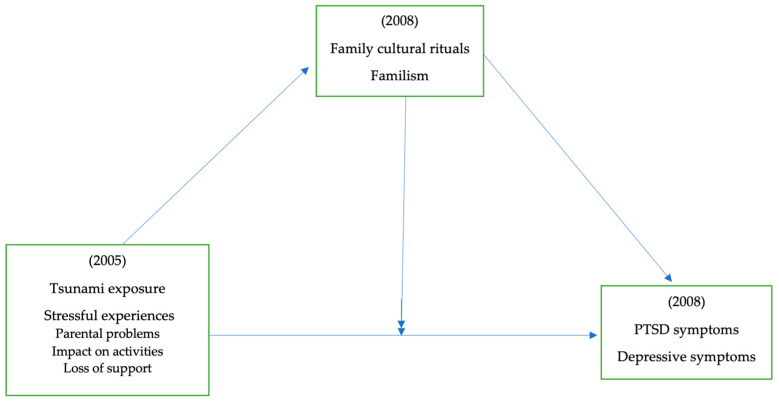
Hypothesized relationships among tsunami exposure, family factors, and mental health outcomes of adolescents.

In the present study, as shown in Figure 2, we hypothesize the following:

- **H1:** For adolescents, greater tsunami exposure at Wave 1 will be associated with more PTSD symptoms and depressive symptoms at Wave 1 and Wave 2 compared to adolescents with lower levels of tsunami exposure at Wave 1;
- **H2:** For adolescents, greater perceived stressful experiences by adolescents (parental problems, impact of parental problems on their activities, and loss of support) at Wave 1 will be associated with more PTSD symptoms and depressive symptoms at Wave 1 and Wave 2 compared to adolescents who experienced lower levels of perceived stressful experiences at Wave 1;
- **H3:** For adolescents, the associations between tsunami exposure and perceived stressful experiences at Wave 1 and PTSD and depressive symptoms at Wave 1 and Wave 2 will be moderated by family cultural rituals and familism.

## 3. Materials and Methods

### 3.1. Sample and Procedure

The data for this study come from two phases of the project conducted by the authors in the village of Polhena examining the mental health of parents and children exposed to the tsunami. Polhena village was directly impacted, and the larger Matara district, within which it resides, had over 50 villages similarly affected. Due to the high exposure and a variation in experiences among families, Polhena village was selected as the site for this current study. Polhena village was not directly affected by the war in Sri Lanka, nor was it exposed to any prior major natural disasters.

In 2005, mother–adolescent dyads were randomly selected from a list of village resident names maintained by the local area government officer. These selected dyads were followed longitudinally from 2005 to 2008. The data were collected through in-person interviews within three months of the tsunami from 235 registered families in Polhena village (Wave 1). Follow-up data were collected via in-person interviews from 160 of the original mother–adolescent dyads in June–July 2008 (Wave 2). Due to post-trauma displacement, the sample mostly included displaced families. Although the loss of about half of the original sample is disappointing, it is important to view this loss in context. We believe that this retention rate is satisfactory as we attempted to follow-up highly mobile disaster-affected families and adolescents over three years.

Attrition analysis on the two waves resulted in no significant differences among those who participated in both waves vs. those who participated in Wave 1 but did not participate in Wave 2. Demographics such as mothers’ age, family size, receipt of public assistance, income, education, and mental illness were similar among participants and non-participants between the two waves. In-home interviews were conducted using 10 trained interviewers. These interviewers were recent graduates with undergraduate degrees currently working as social workers. They were given a two-day training on questionnaire administration by the principal investigators (PIs) of this project. The first day of training focused on data collection methods and in-class exercises, with special attention given to psychiatric symptoms (i.e., PTSD and depression). The second training day provided a session for reviewing and pilot testing interview procedures. Interviewer training focused mainly on collecting data from adolescents and mothers regarding PTSD and depressive symptoms but did not involve case diagnosis. An experienced local psychiatric therapist assisted with the training session and ensured that she would be available for necessary therapy assistance during the interview period. One of the PIs of the project, a Sri Lankan who is an academic in the United States, remained in the area during the survey to coordinate and monitor all research activities and advise the survey team. The PI regularly checked completed questionnaires for data quality and made corrective measures in interview protocol/data collection procedures when necessary. This procedure was followed in both waves of data collection. Authors were on location in Sri Lanka for the recruiting and training of interviewers, project managers, sampling, and data collection. The questionnaire with measures from both Western and Eastern studies were cross-culturally adapted for use in Sri Lanka using a revised Delphi process. The theoretical model and its conceptualizations were operationalized in the questionnaire with socio-culturally meaningful items.

#### Questionnaire

A questionnaire was administered in 2005 and again in 2008 to both adolescents and their mothers. Questionnaires contained subsections on demographic information, tsunami experiences, mental health, physical health, family relationships, marital relationships, parent–child relationships, community relationships, coping strategies, and receipt of government assistance. Questionnaire content detailed information on issues such as PTSD, depressive symptoms, anxiety, stress, physical injuries, lifestyles, substance use, and domestic violence. Additional sections of the questionnaire asked respondents about rituals, traditions, religious practices and beliefs, formal and informal social support, and familism.

### 3.2. Measures

The primary investigator of the project, in collaboration with local mental health professionals, cross-culturally validated the study measures from English to Sinhalese culture and context through an adapted Delphi process [53]. Pilot testing was conducted with five village respondents, at which time translated versions underwent revisions to improve clarity and understanding. Subsequent descriptive statistics and reliability coefficients of study measures illustrated evidence of strong measure reliability.

#### 3.2.1. Tsunami Exposure

A summed composite measure was created using 11 items asking adolescents in Wave 1 about their exposure to the tsunami. These items measured the degree to which the tsunami impacted various aspects of their life. Sample items asked included whether or not they had experienced any of the following as a result of the tsunami: serious injury/threat to own life; death of a parent; destruction of family house; and damage to public property such as school. Each item was scored yes (1) or no (0). Cronbach’s alpha was 0.91. The items used in this measure may represent different non-equivalent experiences in various domains, but as a whole, these capture a cumulative effect on one’s mental health [54].

#### 3.2.2. Impact of Parental Problems on Adolescent Activities

The perceived impact of tsunami-induced parental problems on adolescent activities was measured using two questions from Wave 1: (a) As a result of the tsunami, my parent’s mental health problems have negatively influenced my activities; and (b) As a result of the tsunami, my parent’s increased alcohol consumption has negatively influenced my activities. These were ranked on a Likert scale from 1 (strongly disagree) to 5 (strongly agree). The two items were significantly correlated (*p* < 0.05).

#### 3.2.3. Impact of Tsunami on Adolescent Activities

A summed composite measure was created using three items from Wave 1 asking the following questions: (a) As a result of the tsunami, increased school problems have (negatively) influenced my activities; (b) As a result of the tsunami, the loss of community and friend support has (negatively) influenced my activities; and (c) the tsunami has destroyed my relationships with friends. These were scored on a Likert scale ranging from 1 (strongly disagree) to 5 (strongly agree). Cronbach’s alpha was 0.60.

#### 3.2.4. Adolescent-Perceived Loss of Support

This measure assessed the level of perceived loss of support of adolescents in Wave 1 from various sources as a result of the tsunami. A summed composite measure was created using three items: (a) As a result of the tsunami, the support I received from friends was reduced; (b) As a result of the tsunami, the support I received from the community was reduced; and (c) As a result of the tsunami, the support I received from family was reduced. These were scored on a Likert scale ranging from 1 (strongly disagree to 5 (strongly agree). Cronbach’s alpha for this three-item measure was 0.71.

#### 3.2.5. Family Cultural Rituals

This measure consisted of five items from Wave 2 which asked mothers about their cultural rituals practiced after the tsunami. Sample items included: “how often do you practice Bali and how often do you read your horoscope?” Responses to the questions ranged from 1 (very rarely) to 4 (all the time). Cronbach’s alpha was 0.93, which compares favorably to prior studies utilizing this measure [20,39].

#### 3.2.6. Familism

Mothers responded to 14 items in Wave 2 that assessed cohesion and support among family members [55]. Possible responses to these items ranged from 1 = strongly disagree to 5 = strongly agree. A sample item includes: “How well does each member gets along with the other?” Cronbach’s alpha for this measure was 0.76.

#### 3.2.7. Adolescent Post-Traumatic Stress Disorder (PTSD) Symptoms

Adolescents’ post-traumatic stress disorder (PTSD) symptoms were assessed in both Wave 1 and Wave 2 using 17 diagnostic interview items from the DSM-IV [31]. Items were recoded so response categories were 0 = no (never) and 1 = yes (sometimes or often). These items have yielded acceptable psychometric properties for screening PTSD [6]. Cronbach’s alpha for this measure was 0.87 in Wave 1 and 0.90 in Wave 2. Previous work [6,56,57] has used this measure in populations from Sri Lanka, the Middle East, and Southeast Asia.

#### 3.2.8. Adolescent Depressive Symptoms

A total of 10 items were used for the purpose of assessing adolescent depressive symptomology. These items were derived from the Child Behavior Checklist [58] (Youth Self Report). Sample items included “In the past 7 days, I felt lonely; In the past 7 days, I cried a lot.” Response options for each question ranged from 0 = rarely/none of the time to 3 = most/all the time. Items were summed to yield a range of 0 to 30 for this measure, with evidence of good psychometric properties for use across multiple languages [58]. Cronbach’s alpha was 0.66 for Wave 1 and 0.74 for Wave 2.

### 3.3. Data Analysis

Preliminary analyses consisting of frequencies, descriptive statistics, variable diagnostics, bivariate correlations, means, standard deviations, skewness, and kurtosis of the study variables were conducted. The hypothesized model was tested using Mplus software (version 8.6; [59]) employing structural equation models (SEMs). These analyses provided regression coefficients and test statistics for each path. Chi-square statistics were used to assess the overall fit of the data with our theoretical model [60]. The overall model fit was assessed using multiple indicators including CFI, RMSEA, and Chi-Sq/df. Missing data were accounted for using Full Information Maximum Likelihood procedures [61].

## 4. Results

Table 1 presents descriptive statistics, while Table 2 presents bivariate correlations among the major study variables.

### 4.1. Tsunami Exposure, Stressful Experiences, Adolescent PTSD and Depressive Symptoms

**Figure 3:**
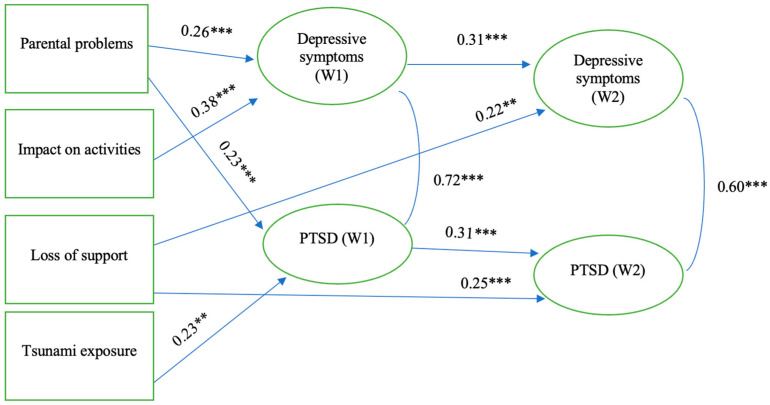
Results of structural equation model for tsunami exposure, stressful experiences, and adolescent PTSD and depressive symptoms. Note: R^2 Dep 1 = 30.1%, R^2 Dep 2 = 16.2%, R^2 PTSD 1 = 9.8%, R^ PTSD 2 = 16.3%; RMSEA = 0.07, Chi-square/df = 3.21, CFI 0.70, ** *p* < 0.01, *** *p* < 0.001, Standardized coefficients are shown.

The results of Model 1 (Figure 3) show that greater perceived parental problems after the tsunami and a greater perceived impact of those problems on adolescent activities significantly increased depressive symptoms in Wave 1 (β = 0.26, *p* < 0.001; β = 0.38, *p* < 0.001, respectively), while greater perceived parental problems after the tsunami and greater tsunami exposure increased PTSD symptoms in Wave 1 (β = 0.23, *p* < 0.001; β = 0.23, *p* < 0.01, respectively). Adolescent-perceived loss of support significantly increased depressive symptoms and PTSD symptoms over time (β = 0.22, *p* < 0.01 and β = 0.25, *p* < 0.001 in Wave 2, respectively). Moreover, depressive symptoms and PTSD symptoms in Wave 1 significantly increased subsequent depressive and PTSD symptoms in Wave 2 (β = 0.31, *p* < 0.001; β = 0.31, *p* < 0.001, respectively), showing moderate stabilities over three years. PTSD symptoms and depressive symptoms were highly correlated across waves. Only adolescent-perceived loss of support showed a long-term influence on PTSD and depressive symptoms at Wave 2. The percentage of explained variance in depressive symptoms at Wave 2 is 16.2%, while PTSD at Wave 2 is 16.3%.

### 4.2. Tsunami Exposure, Stressful Experiences, Family Factors, Adolescent PTSD and Depressive Symptoms: Moderation Model

**Figure 4:**
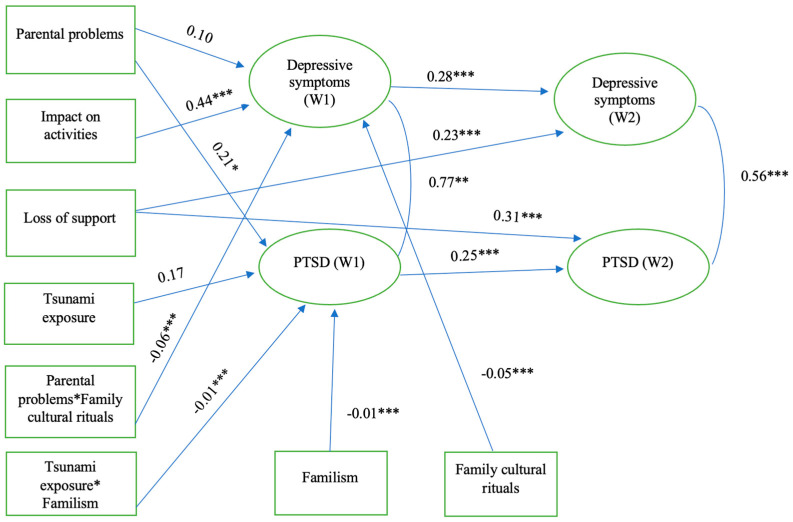
Structural equation model testing moderating effects of parental problems, family cultural rituals, tsunami exposure, and familism. Note: R^2 Dep 1 = 31.7%, R^2 Dep 2 = 14.9%, R^2 PTSD 1 = 13.5%, R^ PTSD 2 = 15.9%, RMSEA = 0.06, Chi-Sq/df = 2.61, CFI = 0.70, * *p* < 0.05, ** *p* < 0.01, *** *p* < 0.001. Predictors and Moderators Mean Centered. Standardized Coefficients are shown. Interaction term constituent paths (direct and interaction terms) are shown.

Model 2 (Figure 4) results show that greater reported impact of parental problems on adolescent activities significantly increases depressive symptoms at Wave 1 (β = 0.44, *p* < 0.001), while greater exposure and greater parental problems significantly increased PTSD symptoms at Wave 1 (β = 0.17, *p* < 0.05; β = 0.21, *p* < 0.05, respectively). Depressive symptoms and PTSD symptoms at Wave 1 significantly increased corresponding depressive symptoms and PTSD symptoms at Wave 2 (β = 0.28, *p* < 0.001; β = 0.25, *p* < 0.001, respectively). Moreover, perceived loss of support by adolescents significantly increased both depressive symptoms and PTSD symptoms at Wave 2 (β = 0.23, *p* < 0.001; β = 0.31, *p* < 0.001). Increased practice of family cultural rituals significantly reduced depressive symptoms at Wave 1 (β = −0.05, *p* < 0.001) and significantly buffered the adverse influence of parental problems on depressive symptoms at Wave 1 (β = −0.06, *p* < 0.001). Familism significantly buffered the adverse influence of tsunami exposure on PTSD symptoms at Wave 1 (β = −0.01, *p* < 0.001). Non-significant paths were not included in the models except for the paths from constituent variables in product terms.

## 5. Discussion

The majority of previous disaster studies have focused overwhelmingly on adults’ mental health consequences related to disaster exposure [6,8,9], with fewer studies focused on the consequences for children and adolescents [62,63]. Research on children and adolescents, particularly in developing countries, to investigate their mental health consequences is insufficient [11]. While studies have tracked poor mental health and PTSD over time following acute-onset disasters, there may be differences when such disasters occur in countries lacking the infrastructure and resources to rapidly recover—thus combining acute and chronic disaster effects [63]. Thus, the present study used a sample of adolescents from Sri Lanka to test the following hypotheses: (1) greater tsunami exposure at Wave 1 will be associated with more PTSD symptoms and depressive symptoms at Wave 1 and Wave 2 compared to adolescents with lower levels of tsunami exposure at Wave 1; (2) greater perceived stressful experiences by adolescents (parental problems, impact of parental problems on their activities, and loss of support) at Wave 1 will be associated with more PTSD symptoms and depressive symptoms at Wave 1 and Wave 2 compared to adolescents who experienced lower levels of perceived stressful experiences at Wave 1; and (3) the associations between tsunami exposure and perceived stressful experiences at Wave 1 and PTSD and depressive symptoms at Wave 1 and Wave 2 will be moderated by family cultural rituals and familism.

Tsunami exposure increased early PTSD symptoms (three months after the tsunami) in adolescents but did not significantly influence early depressive symptoms or later PTSD and later depressive symptoms. This finding is consistent with symptoms stemming from traumatic events, such as serious injuries, property destruction, and witnessed deaths, which help generate PTSD symptoms through cognitive re-experiences, avoidance, and hyperarousal processes [3,31,32]. However, early PTSD symptoms weakly continued over three years, showing that the influence of tsunami exposure on PTSD and associated psycho-cognitive processes did not endure over time. This is similar to what Yule et al. [62] found, where PTSD symptoms were present immediately following a disaster but resolved/did not endure over the long term.

Stressful experiences (parental problems, impact of parental problems on their activities, and loss of support) increased early depressive symptom levels in adolescents. Much of the previous literature considered the adverse effects of the loss of social support on adults’ mental health [64]. The present findings indicate that similar influences exist for adolescents. These influences can include the deprivation of emotional support, such as love, caring, and sympathy, and the erosion of psychological resources, such as a sense of mastery, self-esteem, and competence [65]. This situation is likely aggravated in developing post-disaster communities as youth have diminished access to extra-familial and extra-community support compared to more developed areas with larger networks, including social media.

The impact of parental problems on adolescent activities increased immediate depressive symptoms in adolescents. Parents’ adverse mental health and substance abuse detrimentally affect adolescent social experiences. We suggest this highlights the central role that school plays in the psychosocial well-being of adolescents, for instance, through peer relationships, teacher–student relationships, and school connectedness [66]. Especially in Sri Lanka, where school attendance is compulsory up to 16 years of age, almost all children in their adolescence attend nearby village schools and may be highly attached to their school, teachers, and friends. Losses related to school and school friends are highly stressful, particularly when stemming from parental problems. While immediate depressive symptoms were affected, however, the impact of parental problems on adolescent activities was not significantly associated with later depressive symptoms or initial and later PTSD symptoms.

In contrast, adolescent-perceived loss of support had a long-term influence on both PTSD and depressive symptoms. This finding supports established psychological research on the long-term effects of natural disaster trauma in adolescents [67,68]. The loss of material support may have generated distress feelings, such as hopelessness, helplessness, and fear about the future, in adolescents, largely through structural mechanisms. In Sri Lanka especially, most parents, along with an educationally competitive social environment, encourage children to achieve high educational levels. If adolescents are deprived of the necessary support from sources such as friends, family, and the community, they may be distressed. Perceived support from the aforementioned sources is an important determinant of the long-term mental health outcomes of adolescents, particularly in resource-scarce environments in developing countries.

The results showed that early PTSD and depressive symptoms were highly correlated or comorbid over a three-year period. This finding suggests that a common underlying cause for the prevalence of both PTSD and depressive symptoms other than study predictors may exist. Future research should further investigate the comorbidity between PTSD and depressive symptoms in disaster-exposed adolescents in developing countries. This is particularly important when natural disasters devastate communities already embattled with existing adversities, such as war violence [7].

The mental health effects of tsunami exposure and family loss were reduced by the practice of family cultural rituals and familism. Thus, contextually valid coping methods have been shown to moderate the adverse effects of disasters on youth’s mental illness over time. Cultural coping practices significantly interacted with tsunami-associated family disruptions in moderating their adverse effects on mental health in the long term. The activities associated with cultural coping practices, such as Bali, Thovil, and Puja ceremonies, may have provided spirituality benefits together with inner strength and a belief in a cure through a collectively reinforced belief system of healing [43,44].

Furthermore, the moderating role of family cultural practices can be explained with the perspective of dissociative coping [46,47,48]. Anthropological research has indicated that the cultural categorization of an experience significantly impacts individual outcomes Dissociation is a cross-cultural phenomenon with varying forms and contexts. Dissociative experiences in the context of trauma can be intense, with longer experiences of depersonalization or identity shifts, including spiritualization [69]. In psychiatry, cultural activities such as Bali, Thovil, and Puja ceremonies are seen as functional triggers for neurological mechanisms that produce experiences of dissociation [48,70]. Nevertheless, the results showed that engaging in culturally sanctioned coping strategies helped trauma-exposed adolescents lower their depressive symptoms but not PTSD symptoms. Psycho-cognitive inner processes attributed to cultural practices may operate effectively in a Buddhist religious environment such as Sri Lanka, which is closely linked to the previously discussed cultural practices [71,72].

Another way in which culturally sanctioned coping strategies aid adolescents in relieving distress is through connecting them with social support resources from community members other than their parents who are closely related and integrated into a developing country environment. This support provides an appreciation for reaffirming the community’s cultural beliefs [49]. These psycho-cognitive processes operate effectively in traditional communities because families living in these communities are closely linked through collective, sharing, and supporting social networks at the grassroots level (collectivism) [8,38]. In addition, cultural activities directly decreased early depressive symptoms, likely through compensatory mechanisms. Seemingly, the function of dissociation is only effective for depressive symptoms in adolescents. Future research should further investigate the differential moderating effect of cultural practices related to disaster-exposed adolescents’ PTSD and depressive symptoms and the scope of support it provides.

The results showed that familism significantly moderated the adverse effects of exposure to traumatic events, such as serious injuries, property destruction, and witnessed deaths. Higher levels of support among family members can significantly reduce early PTSD symptoms in adolescents associated with trauma exposure [50]. This protective influence of familism may have operated through cognitive re-experiences, avoidance, and hyperarousal processes [3,31,32]. Thus, familism is a crucial, effective social resource that prevails in traditional societies such as Sri Lanka [23,39]. Fishery families residing in the coastal belt in Sri Lanka who were exposed to the tsunami were particularly characterized by close intra-family links, as all the family members used to collectively engage in family-fishery -elated activities. While findings showed an important connection between familism and PTSD, familism was not significantly linked with depressive symptoms nor did it moderate the link between stressful experiences and depressive symptoms in adolescents. Future research should further investigate the differential moderating effects of familism related to disaster-exposed adolescents’ PTSD and depressive symptoms immediately following a disaster.

The limitations of this study include conceptual, measurement, and analytical considerations. This study could further improve by integrating community factors such as neighborhood, school, and societal support both formal and informal and government assistance available vs. received. While the measures used in this study are self-reported symptoms of illness and socioeconomic experiences, these could be improved by taking more objective assessments from clinicians and verifying socioeconomic measures through multiple respondents. Incorporating nested data from neighborhoods within the village could have allowed us to examine the contextual effect on the outcomes. Family cultural rituals were reported by mothers but could be improved by also including adolescent reports. Moreover, the availability of fathers’ data would allow for a fuller understanding of family influences on adolescent health and analytically allow for a dyadic analytical approach to examine spousal influences on adolescent health.

## 6. Conclusions

The practical uses of this study’s findings are significant. First, these findings contribute to the literature by showing longitudinal relationships between adolescent life disruptions and disaster exposure with mental health problems three years later. Our findings showcase the impact of a natural disaster during the psychosocially and biologically vulnerable adolescent years and how, in a developing country, such early exposure may affect young adults’ mental illness years later. Second, an empirically backed understanding of the contribution to mental health by culturally and socially indigenous practices in Sri Lanka and other developing countries is lacking. This study’s findings highlight the effectiveness of indigenous cultural resources and family practices as protective factors that could be utilized to develop contextually and culturally valid and empirically supported prevention and intervention programs. Such programs would be most effective for trauma-exposed adolescents in developing countries. The knowledge gained from this study can help develop empirically supported prevention and intervention programs to assist adolescents exposed to trauma in developing regions. The findings may also validate the theoretical arguments for socio-cultural coping strategies playing a moderating role in the association between disaster exposure and mental health outcomes among adolescents in the developing world and, specifically, in Sri Lanka. This is an important research direction for regions experiencing historical, ongoing, and complex adversities, such as recurring disasters, war violence, and pandemics, that contribute to long-term generational trauma.

## Figures and Tables

**Table 1:** Descriptive statistics for major study variables.

	Min	Max	Mean	SD
Tsunami Exposure	0	21	11.03	3.47
Parental Problems	1	5	2.11	0.99
Impact on Activities	0	11	6.33	2.44
Loss of Support	2	14	6.82	2.56
Fam Cultural Rituals	0	22	5.60	4.52
Familism	37	70	58.47	7.43
Depression (Wave 1)	0	24	10.38	5.12
Depression (Wave 2)	0	20	4.62	3.97
PTSD (Wave 1)	0	25	10.33	5.26
PTSD 2 (Wave 2)	0	22	6.80	4.84

**Table 2:** Bivariate correlations among major study variables.

	1	2	3	4	5	6	7	8	9
1. Tsunami Exposure									
2. Parental Problems	−0.04								
3. Loss of Support	0.01	0.07							
4. Impact on Activities	−0.01	0.43 **	0.27 **						
5. Fam Cultural Rituals	0.01	−0.16	−0.01	0.02					
6. Familism	0.17 *	−0.22 **	−0.08	−0.19 *	−0.08				
7. Depression (Wave 1)	−0.02	0.38 ***	0.05	0.48 ***	−0.12	−0.06			
8. Depression (Wave 2)	0.10	0.10	0.25 **	0.22 **	−0.08	0.04	0.27 **		
9. PTSD (Wave 1)	0.23 **	0.18 *	0.08	0.22 **	0.03	0.08	0.52 **	0.19 *	
10. PTSD (Wave 2)	0.07	−0.01	0.23 **	0.14	−0.02	−0.03	0.17 *	0.55 ***	0.28

Note: * *p* < 0.05, ** *p* < 0.01, *** *p* < 0.001.

## Data Availability

The data presented in this study are protected by restricted use data sharing agreements with project collaborating organizations and legal bounds for data use within the country of origin. While unable to share data set with readers due to study and legal restrictions, the authors are able to address inquiries involving the data.
